# Massage for rehabilitation after total knee arthroplasty: a systematic review and meta-analysis of randomized controlled trials

**DOI:** 10.1186/s13018-024-04798-6

**Published:** 2024-05-21

**Authors:** Ruinan Chen, Yaoyu Jin, Zhaokai Jin, Yichen Gong, Lei Chen, Hai Su, Xun Liu

**Affiliations:** 1 Zhejiang Chinese Medical University First Clinical Medical College, Hangzhou, 310053 China; 2 The First Affiliated Hospital of Zhejiang, Chinese Medical University, Zhejiang University of Traditional Chinese Medicine First Affiliated Hospital, Hangzhou, 310006 China

**Keywords:** Total knee arthroplasty, Meta-analysis, Rehabilitation, Quality of evidence, Systematic reviews

## Abstract

**Objective:**

This study aimed to evaluate the effectiveness of massage for postoperative rehabilitation after total knee arthroplasty (TKA).

**Data sources:**

The PubMed, Web of Science, EMBASE, Cochrane Library, and China National Knowledge Infrastructure (CNKI) databases were systematically searched from inception to May 2024.

**Study selection:**

Any randomized controlled trials on the use of massage for postoperative TKA rehabilitation were included.

**Data extraction:**

A meta-analysis of outcomes, including postoperative pain, knee range of motion (ROM), postoperative D-dimer levels, and length of hospital stay, was performed. The Cochrane Risk of Bias Assessment Tool was used to assess the risk of bias, and the data for each included study were extracted independently by two researchers.

**Data synthesis:**

Eleven randomized controlled clinical trials with 940 subjects were included. The results showed that compared with the control group, the massage group experienced more significant pain relief on the 7th, 14th and 21st days after the operation. Moreover, the improvement in knee ROM was more pronounced on postoperative days 7 and 14. In addition, the massage group reported fewer adverse events. However, there was no statistically significant difference in the reduction in postoperative D-dimer levels between the patients and controls. Subgroup analysis revealed that massage shortened the length of hospital stay for postoperative patients in China but not significantly for patients in other regions. Nevertheless, the heterogeneity of the studies was large.

**Conclusions:**

Increased massage treatment was more effective at alleviating pain and improving knee ROM in early post-TKA patients. However, massage did not perform better in reducing D-dimer levels in patients after TKA. Based on the current evidence, massage can be used as an adjunctive treatment for rehabilitation after TKA.

**Supplementary Information:**

The online version contains supplementary material available at 10.1186/s13018-024-04798-6.

## Background

Knee osteoarthritis (KOA) is a disease caused by the degeneration of articular cartilage. Knee pain and dysfunction are the main clinical symptoms occurring mostly in middle-aged and older adults [1]. As the disease progresses, it will eventually lead to the loss of knee function, and its expensive treatment costs will burden patients, families, and society [2]. As the world's population ages, more middle-aged and older adults are likely to develop KOA, and one study predicts that by 2032, the proportion of people over 45 years of age with KOA will increase to 15.7% [3].

Total knee arthroplasty (TKA) is widely accepted as an effective treatment for end-stage KOA [4]. After TKA, patients may experience several complications, including pain, swelling, decreased muscle strength, limited joint motion, and even deep vein thrombosis (DVT). These complications seriously affect postoperative rehabilitation and can subsequently seriously affect the recovery of limb function [5]. Therefore, timely and effective postoperative rehabilitation for TKA patients is essential for successful surgery [6].

Massage has a long history of treatment and has evolved throughout the world with different characteristic forms of manipulation, including pressure (gradual downward pressure with fingers or palms on the body surface), rubbing (circular strokes on the body surface), pinching (gentle grasping of soft tissues), vibration (shaking hands to move limbs), and plucking (plucking soft tissues back and forth like strings) [7].

To date, in comparison with traditional rehabilitation methods following TKA such as manual lymphatic drainage and continuous passive movement [8], massage exhibits unique characteristics and has demonstrated favorable efficacy across various disease fields [9, 10]. Similarly, many randomized controlled clinical trials have investigated the effects of massage on postoperative rehabilitation after TKA, but the results are diverse, and there is no clear consensus [11–15]. In addition, no systematic reviews or meta-analyses of these studies have been reported. Therefore, this study collected randomized controlled trials of massage rehabilitation after TKA from different databases and performed a systematic review and meta-analysis to assess the effect of massage on the rehabilitation of patients undergoing TKA surgery.

## Methods

### Study registration

The protocol for this systematic review was registered with PROSPERO (registration number: CRD42023411680). This systematic review is reported based on the Preferred Reporting Items for Systematic Review and Meta-Analysis (PRISMA) 2020 checklist [16].

### Search strategy

Two reviewers (Jin YY, Chen RN) searched the PubMed, Web of Science, EMBASE, Cochrane Library, and China National Knowledge Infrastructure (CNKI) databases for related research from inception to May 2024. Strings such as “Arthroplasty, Replacement, Knee,” “Total Knee Arthroplasty,” “TKA,” “Massage,” “Massage Therapy,” and “Randomized Controlled Trials” were used. The search strategy is detailed in Supplementary Table S1.

### Selection criteria

This study screened the literature according to the principles of the PICOS.

①Participants:

Patients who underwent TKA for the first time. No restrictions were made on patient age, disease course or specific surgical procedure.

② Intervention:

Simple massage therapy or massage combined with routine rehabilitation therapy. In addition, there were no restrictions on the type of massage, duration, frequency, or intensity of the intervention.

③ Comparators:

Massage vs. other treatments, massage & other treatments vs. other treatments, massage vs. placebo.

④ Outcomes:

At least one index of the curative effect of postoperative rehabilitation.

⑤ Study design:

Randomized controlled trials (RCTs).

The literature was excluded if it met any of the following criteria:

① Articles not in English or Chinese; ② Incomplete or repeatedly published literature; ③ Documents required for statistical analysis that could not be integrated or obtained; ④ Full text could not be obtained; ⑤ Revision TKA, single compartment knee arthroplasty; ⑥ Continuous passive motion, manual lymphatic drainage; ⑦ Animal experimental studies, case reports, conference papers, dissertations.

### Data extraction

Two researchers (Jin YY, Chen RN) independently screened all the retrieved literature based on the inclusion and exclusion criteria. An initial literature screening was performed first after reading the titles and abstracts, and then a final screening was performed after carefully reading the full texts of the remaining studies. In the process of literature screening and data extraction, any dissenting opinions were discussed by both parties or handed over to the third-party researcher for decision-making. Two researchers extracted the following information from the final included literature: name of the first author, year of publication, country, patient age and biological sex, sample size, type and duration of intervention, time point of assessment, primary outcomes, and adverse events.

### Quality assessment

The Cochrane Risk of Bias Assessment Tool [17] was used to assess the quality of the included studies. The assessment included seven items: random sequence generation, allocation concealment, blinding of participants and personnel, blinding of outcome assessors, incomplete outcome data, selective reporting, and other biases. Each project risk level is divided into three levels: high risk, low risk, and unclear risk.

### Statistical analysis

Review Manager 5.3 and Stata 16.0 software were used for the statistical analyses. All continuous variables were pooled by mean difference (MD) or standardized mean difference (SMD) with 95% confidence intervals (95% CI). Heterogeneity of the included studies was assessed using the Q statistic and *I*^2^ indices, and meta-analysis was performed using a fixed-effects model when* I*^2^ < 50% and a random-effects model when* I*^2^ > 50%. Differences were also considered statistically significant when *p* < 0.05. Subgroup analyses were performed based on country type to explore potential sources of heterogeneity between studies. Publication bias was assessed using Begg's and Egger's tests (*p* < 0.05, statistically significant difference).

## Results

### Study selection

**Fig. 1:**
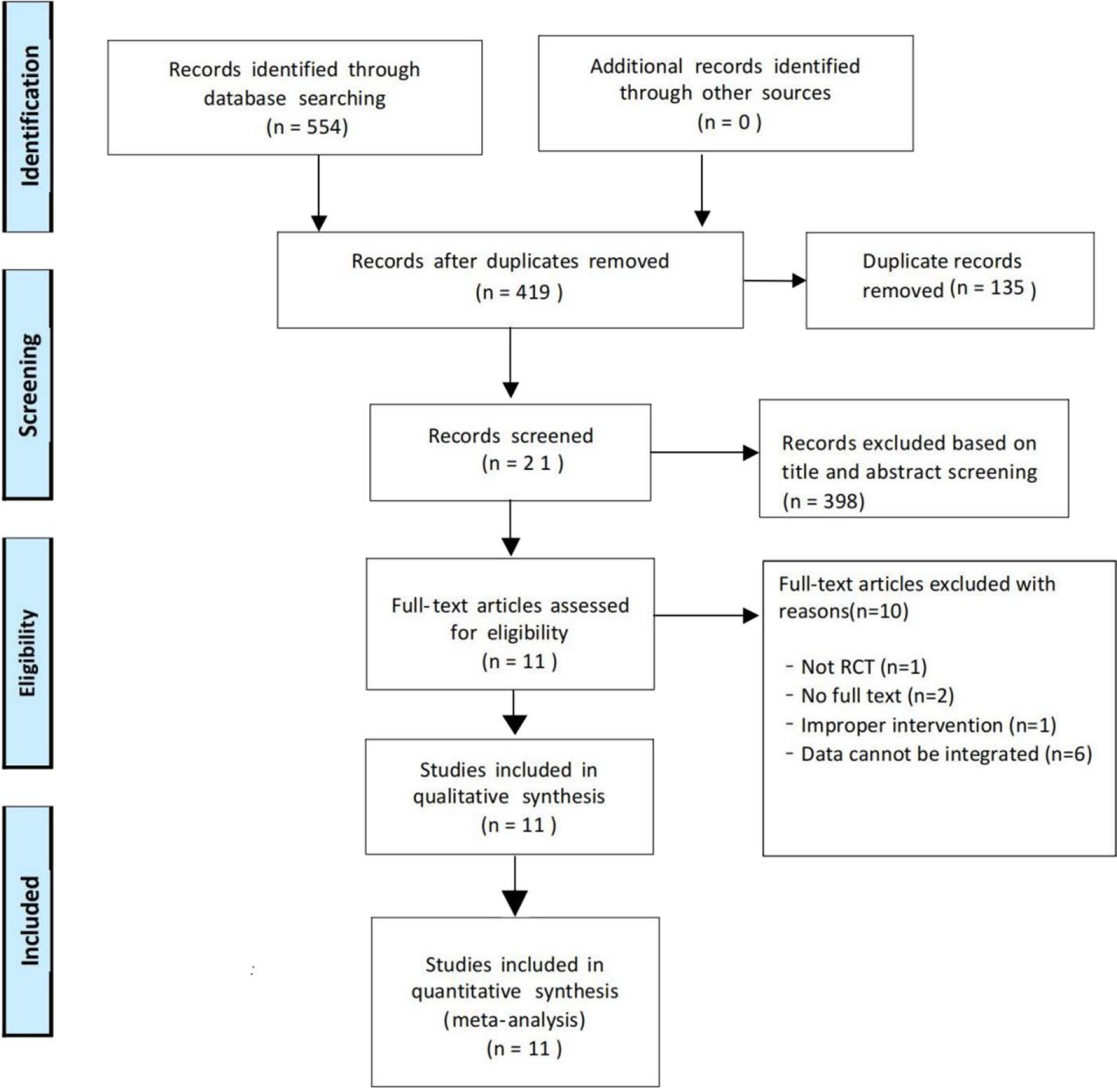
Flowchart of study selection

A total of 554 articles were retrieved by searching each database; 135 articles were eliminated by EndNoteX9 software, 398 were deleted after reading titles and abstracts, and the remaining 21 were retained. After reading the full text, 10 articles were excluded, of which 1 was not an RCT, 2 were not available for full text, 1 did not have the correct intervention, 6 data could not be integrated, and were finally included in 11 articles. The flowchart of the literature selection process is shown in Fig. 1.

A total of 940 patients were included in the 11 articles [11–15, 18–23]. Nine of the studies were from China, while the other two were from Turkey and Japan. The studies, published between 2008 and 2022, ranged in sample size from 30 to 168 people. Except for one study [14] in which the massage method was self-massaged under the guidance of a professional therapist, the massage method in other studies was administered by a professional therapist. And except for one study [20] that used the “massage vs. other treatments” model, the remaining studies used the “massage + other treatments vs. other treatments” model.

In addition, four studies [19, 21–23] did not report the occurrence of adverse events. Table 1 summarizes the characteristics of the included studies.

**Table 1:** Characteristics of the included RCTS

References	Country	Age (Year)		Sample size	Gender(male/female)	
		MG	CG	MG/CG	MG	CG
Fang [11]	China	61.11 ± 7.14	60.06 ± 5.7	19/19	8/11	7/12
Fu [15]	China	61.0 ± 6.4	61.4 ± 6.9	15/15	11/4	10/5
Karaborklu [18]	Turkey	69.3 ± 7.4	67.5 ± 5.01	21/21	4/17	1/20
Tomohiro [14]	Japan	72.5 ± 8.7	73.4 ± 7.1	81/84	16/65	13/71
Xu H [12]	China	68.66 ± 4.80	67.02 ± 5.04	65/63	15/50	12/51
Ma [22]	CHINA	61.24 ± 2.13	61.10 ± 2.94	84/84	22/62	64/60
Wu [19]	China	67.60 ± 8.82	68.77 ± 7.71	30/30	8/22	9/21
Xu [23]	China	68.96 ± 6.32	69.16 ± 7.21	30/30	15/15	13/17
Yuan [20]	China	75.5 ± 1.7	72.3 ± 1.5	36/36	20/16	17/19
Zhao [21]	China	56.7 ± 6.3	43/43	58/28		
Zhao [13]	China	68.56 ± 4.60	67.02 ± 4.86	45/46	10/35	12/34

References	Intervention	Duration/day	Time points for evaluation/day	
	MG	CG		
Fang [11]	Massage & routine rehabilitation	Routine rehabilitation	21D	7D,14D,21D after surgery
Fu [15]	Massage & routine rehabilitation	Routine rehabilitation	15D	15d after surgery
Karaborklu [18]	Massage & Postop Exercises	Postop exercises	2 months	14D,2 months after surgery
Tomohiro [14]	Massage & regular physical therapy	Regular physical therapy	2D	3D after surgery
Xu [12]	Massage & routine analgesia	Routine analgesia	7D	3D,7D after surgery
Ma [22]	Massage & routine rehabilitation	Routine rehabilitation	7D	7D after surgery
Wu [19]	Massage & routine rehabilitation	Routine rehabilitation	14D	14D after surgery
Xu [23]	Massage & routine rehabilitation	Routine rehabilitation	14D	7D,14D after surgery
Yuan [20]	Massage	Routine rehabilitation	21D	21D after surgery
Zhao [21]	Massage & routine rehabilitation	Routine rehabilitation	15D	15D after surgery
Zhao [13]	Massage & rivaroxaban	Rivaroxaban	14D	14d after surgery

References	Main outcome	Adverse events
Fang [11]	ROM of Knee, VAS, HSS, drainage within 2 days after surgery, perimeter of suprapatellar 8 cm within 2 weeks after surgery	CG: one case of DVT was found
Fu [15]	VAS, D-dimer level, total effective rate of rehabilitation after knee arthroplasty, postoperative hospitalization time, time for knee joint function to return to normal, adverse events	CG: three cases of adverse events were found (details unknown)
Karaborklu [18]	ROM of knee, WOMAC, numeric pain-rating scale,10-m walk test,5-times sit to stand test, SF-12	None
Tomohiro [14]	D-dimer level, the incidence of DVT	None
Xu [12]	VAS, ROM of knee, the pressure pain threshold, times of patient-controlled analgesia, additional dose of analgesics, Hamilton anxiety scale score, adverse events	None
Ma [22]	HSS, postoperative hospital stay, SF-36	Not reported
Wu [19]	HSS, D-dimer level	Not reported
Xu [23]	VAS, D-dimer level, postoperative swelling of knee joint	Not reported
Yuan [20]	VAS, HSS, flexion of knee joint, adverse events	None
Zhao [21]	HSS, D-dimer level	Not reported
Zhao [13]	D-dimer level, the incidence of DVT, AKS, common femoral vein stasis index, tenderness threshold, circumference difference of thigh and calf, adverse events	None

MG, Massage group; CG, Control group; VAS, Visual analog scale; ROM, Range of motion; HSS, Hospital for special surgery knee score; WOMAC, Western Ontario and Mcmaster university osteoarthritis index; SF-12, 12-item short form health survey; SF-36, 36-item short form health survey; DVT, Deep venous thrombosis; AKS, American knee society score

### Risk of *bias*

**Fig. 2:**
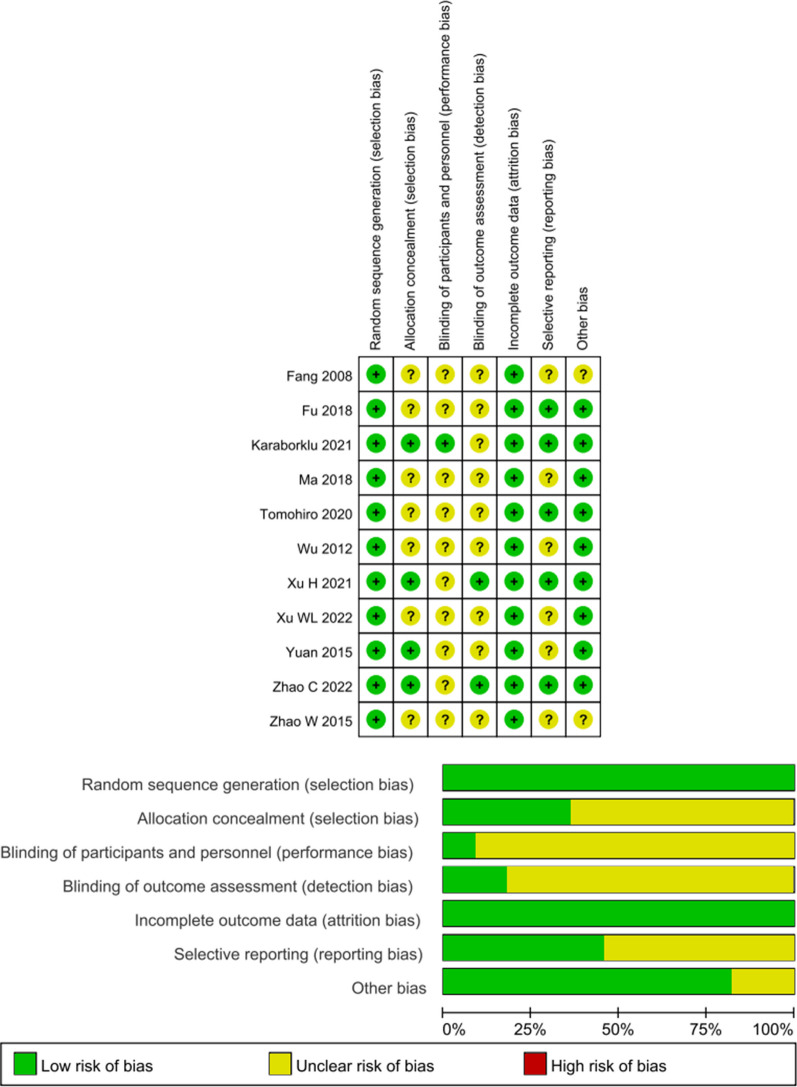
Risk of bias graph

All included studies randomized the allocation of subjects. Nine studies [12–15, 18–20, 22, 23] documented the randomization method in detail, and three [12, 13, 18] of them detailed the allocation concealment process. Two studies [12, 13] were blinded to the outcome assessor. Moreover, only one study [18] described the blinding of subjects and therapists. The detailed results are shown in Fig. 2.

### *Meta*-analysis

#### Postoperative pain

**Fig. 3:**
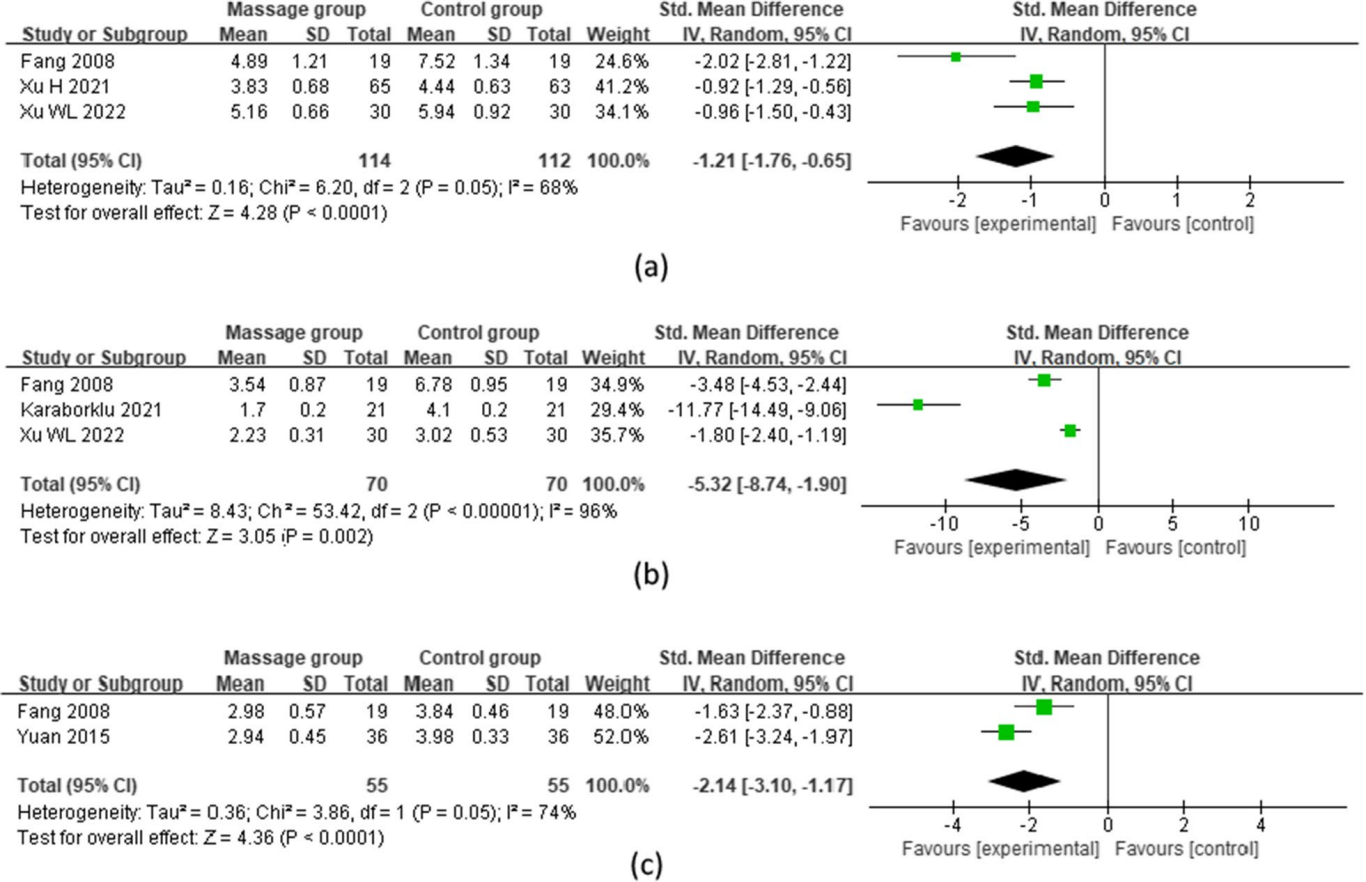
Meta-analysis and forest plot for postoperative pain at different time points. **a**–**c** Pain on postoperative days 7, 14, and 21, respectively

Three studies [11, 12, 23]^, including 226, 140, and 110 patients^, reported pain at 7 days after TKA, three [11, 18, 23] at 14 days after TKA, and two [11, 20] at 21 days after TKA, respectively. A random effects model was used for the meta-analysis. The results showed that the degree of pain in the massage group (MG) was significantly lower than that in the control group (CG) at 7 [MD = -1.21 (95%: − 1.76, − 0.65), *p* < 0.0001, *I*^2^ = 68%];14 [MD = − 5.32 (95%: − 8.74, − 1.90), *p* = 0.02, *I*^2^ = 96%]; and 21 days [MD = − 2.14 (95%: − 3.10, − 1.17), *p* < 0.0001, *I*^2^ = 74%] after surgery (Fig. 3).

#### Knee ROM

**Fig. 4:**
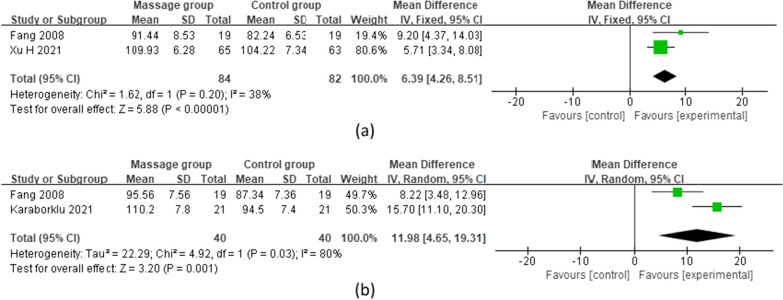
Meta-analysis and forest plot for knee ROM at different periods. **a** Knee ROM on the 7th day after surgery; **b** knee ROM on the 14th day after surgery

Two studies [11, 12] reported knee ROM at 7 days after TKA, including 84 patients in the MG and 82 in the CG. A fixed effects model was used for the meta-analysis. The results showed that the MG improved the knee ROM more than did the CG at 7 days after surgery, and the difference was statistically significant [MD = 6.39 (95%: 4.26,8.51),* p* < 0.00001, *I*^2^ = 38%]. Two studies [11, 18] reported knee ROM at 14 days after TKA, including 40 patients in the MG and 40 in the CG. A random effects model was used for the meta-analysis. The results showed that the MG improved the knee ROM more than did the CG at 14 days after surgery, and the difference was statistically significant [MD = 11.98 (95%: 4.65, 19.31), *p* = 0.001, *I*^2^ = 80%] (Fig. 4).

#### Postoperative D-dimer levels

**Fig. 5:**
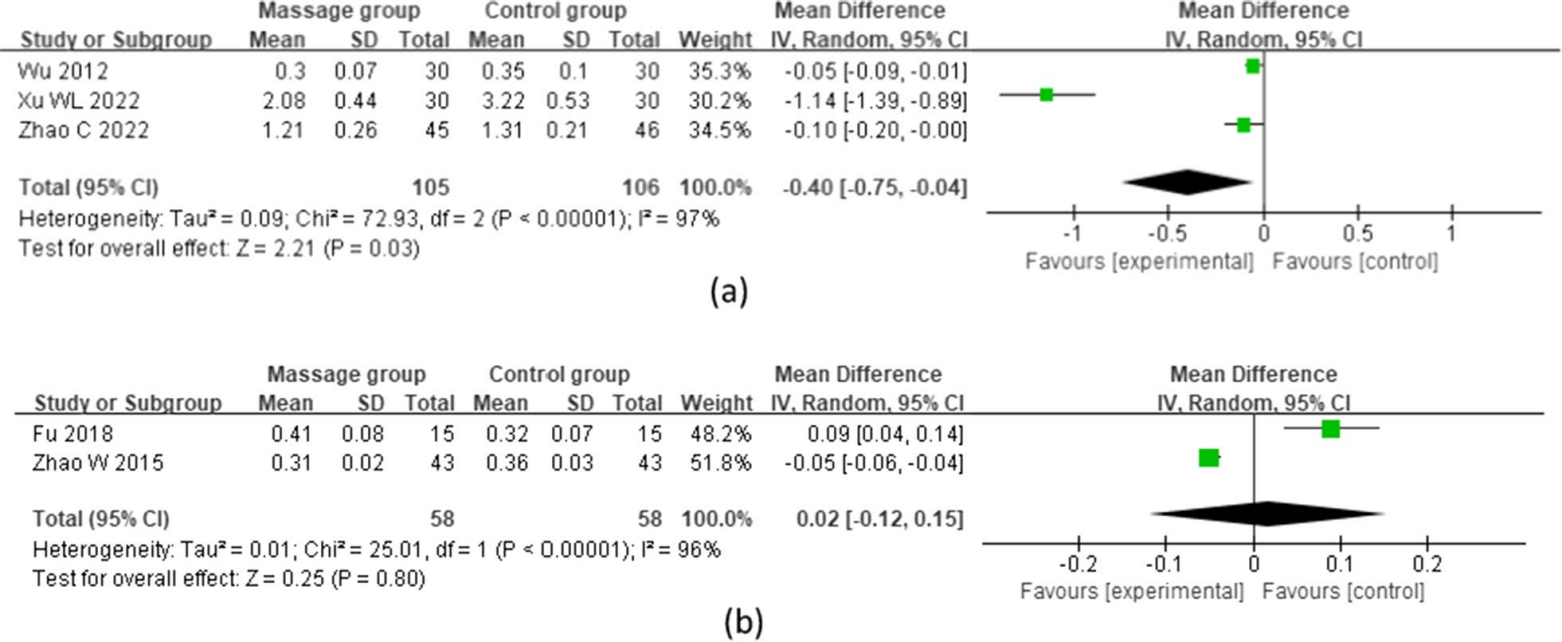
Postoperative D-dimer levels at different time points. **a** D-dimer level on the 14th day after surgery. **b** D-dimer level on the 15th day after surgery

Three studies [13, 19, 23] reported D-dimer levels at 14 days after TKA, including 105 patients in the MG and 106 in the CG. A random effects model was used for the meta-analysis. The results showed that the D-dimer level in the MG decreased more significantly than that in the CG at 14 days after surgery, and the difference was statistically significant [MD = -0.40 (95%: -0.75, -0.04), *p* = 0.03, *I*^2^ = 97%]. Two studies [15, 21] reported D-dimer levels 15 days after TKA, including 58 patients in the MG and 58 in the CG. A random effects model was used for the meta-analysis. However, there was no significant difference in D-dimer levels between the massage and control groups at 15 days after surgery [MD = 0.02 (95%: − 0.12,0.15), *p* = 0.80, *I*^2^ = 96%] (Fig. 5).

#### Length of hospital stay

**Fig. 6:**
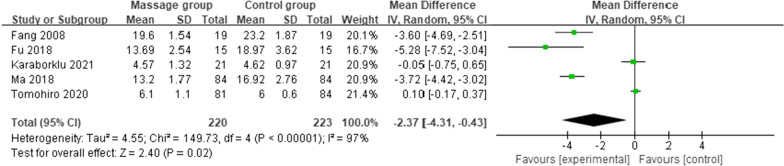
Length of hospital stay

Five studies [11, 14, 15, 18, 22] reported the length of hospital stay after TKA, including 220 patients in the MG and 223 in the CG. A random effects model was used for the meta-analysis. The results showed that the length of hospital stay was significantly shorter in the MG than in the CG [MD = − 5.32 (95%: − 8.74, − 1.90), *p* = 0.002, *I*^2^ = 96%] (Fig. 6).

### Subgroup analyses

**Fig. 7:**
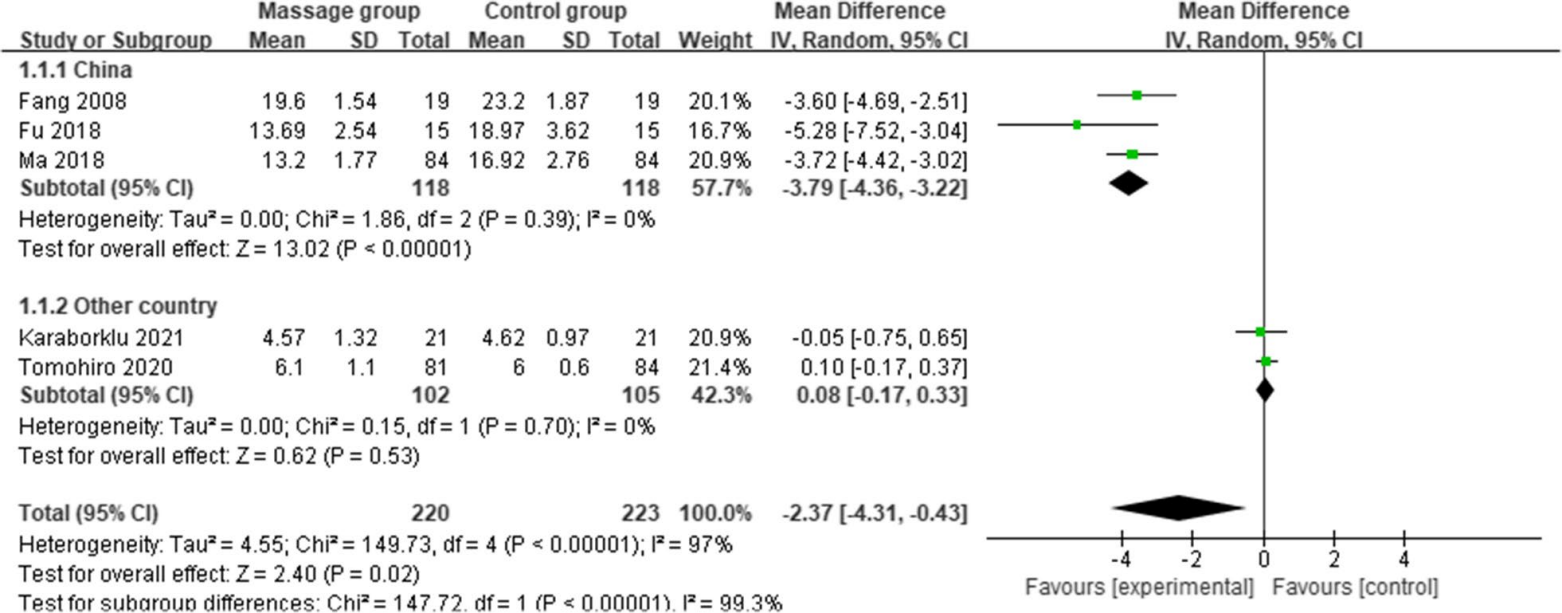
Subgroup analysis of length of hospital stay

Due to the small number of studies included for the remaining outcomes, we performed subgroup analyses only for the outcome of length of hospital stay. To explore the sources of heterogeneity, we divided the analysis into two subgroups, China and other countries, according to country type. The results showed that massage treatment shortened the length of hospital stay for TKA patients in China [MD = − 3.79 (95%: − 4.36, 3.22), *p* < 0.00001, *I*^2^ = 0%] but not for TKA patients in other countries [MD = 0.08 (95%: − 0.17, 0.33), *p* = 0.53, *I*^2^ = 0%], and the heterogeneity between the two subgroups was significantly lower, confirming the country type as a source of heterogeneity (Fig. 7).

In addition, according to the Cochrane Handbook [24], sensitivity analysis was not performed in this study due to the small amount of literature included for each outcome.

### Publication *bias*

In this study, we examined publication bias using Begg's and Egger's tests. The results showed potential publication bias in the outcome of postoperative day 14 pain according to Egger's test (*p* = 0.049), and no evidence of significant publication bias was found in the remaining included studies. (*p* > 0.05). In addition, Egger's test could not be performed for pain at postoperative day 21, knee ROM at postoperative day 7, knee ROM at postoperative day 14, or D-dimer level at postoperative day 15 because only two studies were included for each outcome. (Supplementary Table S2).

### Adverse events

Seven studies [11–15, 18, 20] reported adverse events. In one study [11], one patient in the CG was reported to have DVT, and another study [15] reported adverse events in three patients in the CG, but the details were not available. No adverse events were reported in the remaining studies.

## Discussion

According to the evidence from this study, massage has certain therapeutic effects on reducing pain and improving ROM in patients with early-stage TKA, which may help patients leave the hospital earlier and return to normal life. At the same time, massage is safe and reliable. However, massage does not reduce the level of D-dimer in patients after surgery. The mechanism of massage is related to the following factors.

It has been shown that 10–34% of patients experience severe pain after TKA, often leading to chronic pain if not effectively treated [25, 26]. Massage can reduce inflammatory cell infiltration and tissue necrosis in pain mechanisms, as well as the release of neuropeptides, thus preventing chronic pain caused by the constant sensitization of pain-sensing nerves [27, 28].

Furthermore, we believe that these beneficial effects of massage are related to the complex interaction between the therapist and patient. Patients receiving massage receive more care and attention, which to some extent eliminates postoperative anxiety, thereby achieving pain relief. These effects cannot be achieved through routine rehabilitation or medication alone [29].

Quadriceps muscle strength plays an important role in knee function, but most patients fear exercise due to postoperative pain, leading to muscle wasting and decreased muscle strength, which in turn affects the recovery of knee ROM. In contrast, massage has been shown to improve muscle strength and increase knee ROM and stability [30, 31]. The results of our statistical analysis showed that massage improved knee ROM in the early postoperative period after TKA.

D-dimer levels are clinically important indicators for monitoring the occurrence of lower extremity DVT after TKA [32]. There is evidence that massage can prevent DVT, but most studies strongly recommend that massage should be combined with anticoagulants, compression stockings, and pneumatic compression therapy to be effective for DVT prevention [33]. Our results suggest that the addition of massage therapy to routine rehabilitation was ineffective in reducing D-dimer levels after TKA.

The results of our subgroup analysis of the length of hospital stay showed that massage treatment was able to shorten the length of hospital stay for TKA patients in China but not for TKA patients in other countries. It is speculated that this may be related to the different types of massage manipulation and hospital management systems used in different regions.

The present study has several limitations. First, although we conducted as comprehensive a search as possible with no commercial interest involved, publication bias detection may indicate potential publication bias, indicating that some studies published in the gray literature may have been overlooked. Second, most of the included studies were conducted in China, which largely limits the generalizability of massage in post-TKA rehabilitation. The results should be further validated through multicenter and diverse clinical trials. Third, in terms of research design, the nature of massage made it difficult to implement the double-blind method in most studies, reducing the quality of the final evidence and resulting in the quality of the included studies being mostly low to moderate. Therefore, more rigorous scientific design, improved randomization, allocation concealment and blinding methods should be implemented in future research to improve the quality of evidence.

Fourth, muscle strength, knee swelling and quality of life scores are also important in assessing the outcome of TKA, and future studies should improve the collection of these indicators. In addition, in terms of adverse events, the included studies did not report the severity of adverse events. Therefore, the use of a special scale to evaluate the severity of adverse events or adverse events for statistical analysis is also an important direction for subsequent design.

Fifth, most meta-analyses showed great heterogeneity (*I*^2^ > 50%), which was strongly associated with different types, durations, frequencies or intensities of massage in the included studies because massage itself is a regional, individual, diverse characteristic of the treatment. Therefore, it is necessary to develop a set of standardized massage methods for post-TKA active clinical research.

Finally, due to the small number of studies included for each outcome, sensitivity analyses were not performed, which may have contributed to the lack of robustness of the results of the meta-analyses.

## Conclusion

The present study is the first systematic review and meta-analysis to evaluate the efficacy of massage on postoperative rehabilitation in patients undergoing TKA. We conclude that low- to moderate-quality evidence suggests that massage can reduce early pain and improve early knee mobility in patients after TKA, but massage does not reduce D-dimer levels in patients after TKA. Therefore, based on the current evidence, we believe that massage can be used as an adjunctive treatment for postoperative rehabilitation after TKA. However, larger and higher-quality trials are needed to confirm these results in the future.

### Supplementary Information

Supplementary Material 1.

## Data Availability

The datasets used and/or analyzed during the current study are available from the corresponding author on reasonable request.

## Abbreviations

KOA: Knee osteoarthritis
ROM: Knee range of motion
TKA: Total knee arthroplasty
DVT: Deep vein thrombosis
PICOS: Participants, intervention, comparators, outcomes and study design
MD: Mean difference
SMD: Standardized mean difference
CI: Confidence interval
I2: Heterogeneity
