# Supplementary material for: Massage for rehabilitation after total knee arthroplasty: a systematic review and meta-analysis of randomized controlled trials
Source: J Orthop Surg Res. 2024 May 21;19:307. doi: 10.1186/s13018-024-04798-6 (PMC11110294; doi:10.1186/s13018-024-04798-6)
Supplement: Supplementary file 1 — Supplementary Material 1. [file 13018_2024_4798_MOESM1_ESM.docx]

Supplementary Material

**Table S1-Search strategy used**

| **PubMed** | #1 ((((((((((((("Arthroplasty, Replacement, Knee"[Mesh]) OR (total knee arthroplasty[Title/Abstract])) OR (Knee Replacement, Total[Title/Abstract])) OR (Total Knee Replacement[Title/Abstract])) OR (Replacement, Total Knee[Title/Abstract])) OR (Total Knee Arthroplasty[Title/Abstract])) OR (Arthroplasty, Total Knee[Title/Abstract])) OR (Knee Arthroplasty, Total[Title/Abstract])) OR (Replacement Arthroplasties, Knee[Title/Abstract])) OR (Knee Replacement Arthroplasty[Title/Abstract])) OR (Knee Replacement Arthroplasties[Title/Abstract])) OR (Arthroplasty, Knee Replacement[Title/Abstract])) OR (Arthroplasties, Replacement, Knee[Title/Abstract])) OR (TKA[Title/Abstract])  44,448  #2 ((((((((((((((((("Massage"[Mesh] OR "Musculoskeletal Manipulations"[Mesh]) OR (Massage Therapy[Title/Abstract])) OR (Massage Therapies[Title/Abstract])) OR (Therapies, Massage[Title/Abstract])) OR (Therapy, Massage[Title/Abstract])) OR (Manipulations, Musculoskeletal[Title/Abstract])) OR (Manipulation Therapy[Title/Abstract])) OR (Manipulative Therapies[Title/Abstract])) OR (Manipulative Therapy[Title/Abstract])) OR (Therapies, Manipulative[Title/Abstract])) OR (Therapy, Manipulative[Title/Abstract])) OR (Therapy, Manipulation[Title/Abstract])) OR (Manipulation Therapies[Title/Abstract])) OR (Therapies, Manipulation[Title/Abstract])) OR (Manual Therapies[Title/Abstract])) OR (Manual Therapy[Title/Abstract])) OR (Therapies, Manual[Title/Abstract])) OR (Therapy, Manual[Title/Abstract])  22,426  #3 (randomized controlled trial[pt] OR controlled clinical trial[pt] OR randomized[tiab] OR placebo[tiab] OR drug therapy[sh] OR randomly[tiab] OR trial[tiab] OR groups[tiab] NOT (animals [mh] NOT humans [mh]))  5,278,509  #4 #1 AND #2 AND #3 111 |
| --- | --- |
| **Embase** | #1 'total knee arthroplasty'/exp  38,229  #2 'arthroplasty, replacement, knee':ab,ti OR 'knee replacement, total':ab,ti OR 'total knee replacement':ab,ti OR 'replacement, total knee':ab,ti OR 'total knee arthroplasty':ab,ti OR 'arthroplasty, total knee':ab,ti OR 'knee arthroplasty, total':ab,ti OR 'replacement arthroplasties, knee':ab,ti OR 'knee replacement arthroplasty':ab,ti OR 'knee replacement arthroplasties':ab,ti OR 'arthroplasty, knee replacement':ab,ti OR 'arthroplasties, replacement, knee':ab,ti OR tka:ab,ti  39,739  #3 #1 OR #2 48,631  #4 'massage'/exp  19,298  #5 massage:ab,ti OR 'musculoskeletal manipulations':ab,ti OR 'massage therapy':ab,ti OR 'massage therapies':ab,ti OR 'therapies, massage':ab,ti OR 'therapy, massage':ab,ti OR 'manipulations, musculoskeletal':ab,ti OR 'manipulation therapy':ab,ti OR 'manipulative therapies':ab,ti OR 'manipulative therapy':ab,ti OR 'therapies, manipulative':ab,ti OR 'therapy, manipulative':ab,ti OR 'therapy, manipulation':ab,ti OR 'manipulation therapies':ab,ti OR 'therapies, manipulation':ab,ti OR 'manual therapies':ab,ti OR 'manual therapy':ab,ti OR 'therapies, manual':ab,ti OR 'therapy, manual':ab,ti  22,327  #6 #4 OR #5 31,408  #7 'randomized controlled trial'/exp OR 'controlled clinical trial'/exp OR 'randomized':  ti,ab OR 'placebo':ti,ab OR 'drug therapy':lnk OR 'randomly':ti,ab OR 'trial':ti,ab OR  'groups':ti,ab 9,344,627  **#**8 #3 AND #6 AND #7 47 |
| **Cochrane Library** | #1 MeSH descriptor: [Arthroplasty, Replacement, Knee] explode all trees 3,748  #2 (total knee arthroplasty or Knee Replacement, Total or Total Knee Replacement or Replacement, Total Knee or Total Knee Arthroplasty or Arthroplasty, Total Knee or Knee Arthroplasty, Total or Replacement Arthroplasties, Knee or Knee Replacement Arthroplasty or Knee Replacement Arthroplasties or Arthroplasty, Knee Replacement or Arthroplasties, Replacement, Knee or TKA):ti,ab,kw(Word variations have been searched)  9,846  #3 #1 OR #2 9,913  #4 MeSH descriptor: [Massage] explode all trees  1,751  #5 MeSH descriptor: [Musculoskeletal Manipulations] explode all trees 3,877  #6 (Massage Therapy or Massage Therapies or Therapies, Massage or Therapy, Massage or Manipulations, Musculoskeletal or Manipulation Therapy or Manipulative Therapies or Manipulative Therapy or Therapies, Manipulative or Therapy, Manipulative or Therapy, Manipulation or Manipulation Therapies or Therapies, Manipulation or Manual Therapies or Manual Therapy or Therapies, Manual or Therapy, Manual):ti,ab,kw(Word variations have been searched) 25,690  #7 #4 OR #5 OR #6  26,448  #8 #3 AND #7 159 |
| **Web of Science** | #1 TS=(Arthroplasty, Replacement, Knee OR total knee arthroplasty OR Knee Replacement, Total OR Total Knee Replacement OR Replacement, Total Knee OR Total Knee Arthroplasty OR Arthroplasty, Total Knee OR Knee Arthroplasty, Total OR Replacement Arthroplasties, Knee OR Knee Replacement Arthroplasty OR Knee Replacement Arthroplasties OR Arthroplasty, Knee Replacement OR Arthroplasties, Replacement, Knee OR TKA) 73,475  #2 TS=(Massage OR Musculoskeletal Manipulations OR Massage Therapy OR Massage Therapies OR Therapies, Massage OR Therapy, Massage OR Manipulations, Musculoskeletal OR Manipulation Therapy OR Manipulative Therapies OR Manipulative Therapy OR Therapies, Manipulative OR Therapy, Manipulative OR Therapy, Manipulation OR Manipulation Therapies OR Therapies, Manipulation OR Manual Therapies OR Manual Therapy OR Therapies, Manual OR Therapy, Manual)  177,469  #3 TS=(randomized controlled trial OR randomized)  1,456,996  #4 #1 AND #2 AND #3  109 |
| **CNKI** | （（主题：手法）OR（主题：推拿））AND（（主题：全膝关节置换) OR（主题：人工全膝关节置换）OR（主题：全膝人工关节置换）OR（主题： TKA））  128 |

**Table S2-**Assessment of publication bias.

| Outcomes | N | Begg' s test | Egger's test |
| --- | --- | --- | --- |
| Postoperative pain (7 d) | 3 | 0.296 | 0.31 |
| Postoperative pain (14 d) | 3 | 0.296 | 0.049 |
| Postoperative pain (21 d) | 2 | 1 | NA |
| Knee ROM (7d) | 2 | 1 | NA |
| Knee ROM (14d) | 2 | 1 | NA |
| Postoperative D-dimer level (14d) | 3 | 0.296 | 0.297 |
| Postoperative D-dimer level (15d) | 2 | 1 | NA |
| Length of hospital stay | 5 | 0.462 | 0.119 |

N: number of studies; NA: not available.
